# Weakly solvating electrolytes: a solvation-centric paradigm for rechargeable metal batteries

**DOI:** 10.1039/d5sc06221g

**Published:** 2025-10-16

**Authors:** Mehdi Karbak, Kyungmin Yim, Ying Shirley Meng, Yuyan Shao, Wu Xu

**Affiliations:** a Energy and Environment Directorate, Pacific Northwest National Laboratory Richland Washington 99354 USA wu.xu@pnnl.gov; b Energy Storage Research Alliance, Argonne National Laboratory Lemont Illinois 60439 USA; c Pritzker School of Molecular Engineering, University of Chicago Chicago Illinois 60637 USA; d Department of NanoEngineering, University of California San Diego La Jolla California 92093 USA

## Abstract

Electrolyte design has long followed a solvation-first paradigm that prioritizes solvents capable of maximizing salt dissociation and ionic conductivity, while treating interfacial degradation and rheological limitations as secondary constraints. Although this approach has enabled significant progress in lithium-ion and sodium-ion batteries, it inherently favors solvent-dominated solvation structures that destabilize reactive metal interfaces. Weakly solvating electrolytes (WSEs) offer a fundamentally different strategy. By using solvents with intrinsically low donor strength and minimal electrostatic affinity for cations, WSEs suppress cation–solvent coordination and promote anion-rich solvation shells without relying on salt superconcentration. This shift lowers desolvation barriers, redirects interfacial decomposition pathways, and supports the formation of inorganic-rich, stable interphases. In this review, we discuss the molecular and solvation criteria that distinguish WSEs from conventional and concentrated or locally concentrated electrolyte systems, examine their implementation across different chemistries, and identify unresolved design challenges. WSEs are presented not as a niche formulation, but as a solvation-centric framework for rethinking electrolyte function in metal battery technologies.

## Abstract 2

This review establishes weakly solvating electrolytes as a solvation-centric design paradigm, integrating DN/*ε*/ESP descriptors to explain mechanisms, guide solvent selection, and unify strategies across Li, Na, K, Zn, and Mg batteries.

## Graphical abstract

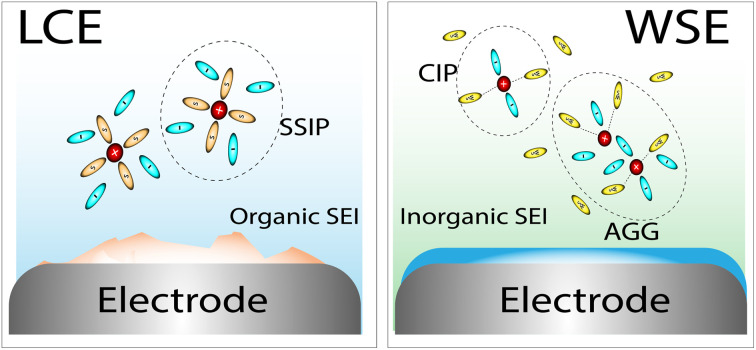

## 1. Introduction

Battery research over the past decade has followed a clear trajectory of discovery and innovation, reaching several milestones in energy and power densities and representing the climax of energy storage development. From material design,^1^ electrolyte formulation^2^ to cycling protocols and safety measurements,^3^ each research path converges toward efficient, stable and safe energy storage. Central to this advancement has been the pursuit of electrolyte systems that simultaneously offer high ionic conductivity and interfacial stability, properties governed primarily by the interplay between the solvent and the salt. In the early stages of electrolyte engineering, the prevailing approach focused on selecting solvents with high dielectric constants (*ε*) and donor numbers (DN).^4^ These properties facilitate salt dissociation and ion mobility by stabilizing charged species, thereby ensuring reliable ion transport. Such solvents, like cyclic and linear carbonates, ensure good ionic transport by stabilizing free ions, leading to a solvation structure dominated by solvent-separated ion pairs (SSIPs) in conventional low concentration electrolytes (LCEs). In SSIPs, the cation is surrounded exclusively by solvent molecules, with the anion separated by at least one solvent layer. This solvation structure results in high mobility of charge carriers and relatively fast ion transport, which made these systems a technological success in the context of early lithium (Li)-ion battery design.^5^ However, SSIP-rich electrolytes introduce critical limitations, especially when paired with reactive alkaline metal anodes such as Li or sodium (Na). The solvent-rich solvation shell favors solvent decomposition at the metal anode surface, leading to unstable, organic-rich solid electrolyte interphases (SEIs), poor coulombic efficiency (CE), and parasitic reactions.^6^ These instabilities also limit the voltage window, temperature tolerance, and long-term cycling performance.

The realization that solvation structure governs interfacial chemistry and electrolyte stability has marked a conceptual shift in electrolyte design. Rather than relying solely on solvent selection for achieving solubility and conductivity, researchers began to focus on tuning the coordination environment of the cation.^7^ By varying the salt-to-solvent ratio and strategically choosing solvent–salt combinations, it became possible to deliberately tailor the solvation structure and unlock new electrolyte behaviors with improved performance characteristics.^8^ This rationale supported the development of high-concentration electrolytes (HCEs). In contrast to conventional formulations where cations and anions are largely dissociated, HCEs use salt-rich compositions to reduce the amount of free solvent molecules available.^9^ As a result, anions are forced into the primary solvation shell, giving rise to contact ion pairs (CIPs) and aggregates (AGGs) and modifying the solvation dynamics.^10^ In these systems, the cation is coordinated by both solvent molecules and anions. The presence of anions in the inner solvation shell has a profound effect on interfacial reactions, shifting the decomposition pathway toward the formation of robust, inorganic-rich SEI on anode and cathode electrolyte interphase (CEI) on cathode. This improves the chemical stability of the electrolyte and suppresses undesirable side-reactions to the electrodes. However, the high salt content increases viscosity, lowers ionic mobility, and reduces wettability posing challenges for electrode infiltration and rate performance.^11^ Additionally, the large amount of expensive salt required makes such systems economically and practically challenging.

To overcome these drawbacks, the concept of localized high-concentration electrolytes (LHCEs) was introduced. LHCEs preserve the advantageous solvation structure of HCEs while mitigating their limitations. This is achieved by diluting the HCE with a non- or weakly coordinating co-solvent, often referred to a diluent. These diluents do not or limitedly enter the primary solvation shell but effectively reduce the viscosity, enhance wettability, and improve the processing characteristics of the electrolyte.^12^ The core principle behind LHCEs lies in structural decoupling, where the local solvation structure remains similar to that of HCEs, while the bulk properties resemble those of more diluted systems. This allows for stabilizing CIPs and AGGs without sacrificing any macroscopic properties. Notably, LHCEs have enabled enhanced cycle life in Li-metal and Na-metal batteries and have facilitated compatibility with high-voltage cathodes and advanced electrode architectures.^13,14^ However, LHCEs are not without limitations. The selection of diluents is critical: they must be inert under operating conditions and must not disrupt the solvation structure. Additionally, the conductivity of LHCEs remains low and sensitive to the nature and proportion of the diluent, as these solvents typically have poor salt solvating power. In some systems, phase separation, volatility, or chemical incompatibility with electrode materials may also present challenges.^15^ Nonetheless, LHCEs represent a strategic evolution in electrolyte design, balancing interfacial chemistry and bulk performance by leveraging solvation structure engineering.

**Fig. 1:**
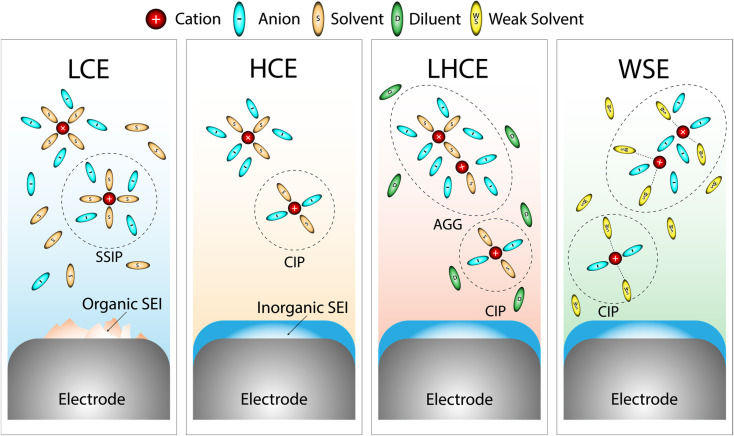
Schematic illustration representing the solvation structures and SEI formation properties of different electrolyte types: LCE, HCE, LHCE and WSE.

Recently, an alternative electrolyte design philosophy emerged from a fundamental reconsideration of solvation itself. In both conventional and concentrated or locally concentrated systems, the prevailing strategy prioritizes solvents that efficiently dissociate salts, maximizing ionic conductivity and mobility, then addressing interfacial or rheological challenges as downstream constraints. This solvation-first paradigm, while effective, imposes structural limitations. A distinct approach departs from this model by employing weakly solvating solvents (WSSs), molecular systems characterized by low DNs and limited electrostatic interaction with cations.^16^ In such media, the solvent contributes minimally to cation coordination, promoting anion-rich solvation structures even at moderate salt concentrations. This enables the formation of weakly solvating electrolytes (WSEs), where the primary solvation shell is dominated by anions rather than solvent molecules as in conventional LCEs.^17^ Instead of enforcing this coordination environment through high salt content, as in HCEs, WSEs achieve it intrinsically through solvent selection. Fig. 1 represents a clear comparison between different electrolyte designs based on the solvation structure and SEI formation properties. The WSE design logic redefines the electrolyte not as a medium to maximize transport, but as a molecular framework to control solvation structure as the governing constraint. Interest in WSEs has been explored in several studies and reviews, which have highlighted their potential in addressing interfacial instabilities and expanding electrolyte design space.^18–20^

The WSE framework has proven applicable across a range of metal battery chemistries, like Li,^21^ Na,^22^ zinc (Zn),^23^ magnesium (Mg),^24^ and under demanding operating conditions such as high voltage^25^ or low temperature.^26^ Beside enabling improved interfacial stability and metal reversibility, WSEs offer a unique platform to interrogate fundamental processes such as cation desolvation, ion transport, and interphase formation.^16,27,28^ Their distinct solvation environment alters both the thermodynamics and kinetics of interfacial reactions, providing mechanistic clarity into phenomena that are often obscured in conventional systems. In this review, we define the emerging class of WSEs, dissect the molecular and solvation characteristics that differentiate them, and examine key studies that have leveraged WSEs to elucidate critical aspects of battery operation. Finally, we identify the current limitations of WSE design and propose pathways to expand their applicability across chemistries, interfaces, and device architectures.

## 2. Fundamentals of WSEs

### 2.1. Definition and key characteristics of WSEs

The terminology surrounding WSEs remains imprecise. In several studies, formulations such as LHCEs have been mischaracterized as WSEs, simply due to the emergence of anion-rich solvation environments. However, this conflation overlooks a fundamental distinction: in LHCEs, almost all solvating solvent molecules are coordinated with cations and there are ideally no free solvating solvent molecules, thus solvents with high solvating capability are required though the added diluent molecules may reduce this solvating capability through the dipole–dipole interactions with the solvating solvent molecules. While in WSEs, such solvation structures arise from the intrinsic weakness of the solvent's interaction with the cation compared to the anion's interactions, not from salt excess. Therefore, a WSE is best defined as an electrolyte in which the coordination between metal cations and solvent molecules is deliberately suppressed at the molecular level. This is typically achieved by employing solvents with low DN, weak polarity, and limited electrostatic affinity for the cation.^16^ In such systems, the anion gains a competitive advantage in occupying the primary solvation shell, even at moderate salt concentrations.

The consequences of weak solvation are both interfacial and transport related. Weakened cation–solvent interactions reduce desolvation barriers, facilitating more efficient charge transfer.^27^ Simultaneously, the suppression of solvent coordination limits its reductive decomposition often leading to SEIs dominated by anion-derived, inorganic components. These structural features distinguish WSEs not by their formulation or performance, but by their governing solvation chemistry. Quantitative thresholds are emerging as DN below ∼10 kcal mol^−1^ and *ε* below ∼5, which are normally associated with weak solvation environments, though variations exist depending on the salt and system.

To complement these descriptors, WSEs can also be diagnosed in terms of their solvation spectrum, using quantitative ranges and experimental observables. In practice, the weakly solvating regime is best understood as a spectrum rather than a sharp boundary, and several measurable indicators can be used to diagnose it across different chemistries. Molecular dynamics and spectroscopic analyses generally reveal that WSEs exhibit anion participation exceeding ∼40–60% of the first solvation shell at salt concentrations ≤1 M, in contrast to conventional dilute electrolytes where solvent ligands dominate. Electrochemical benchmarks further suggest that cation–solvent desolvation free energies are typically reduced by ∼20–40 kJ mol^−1^ relative to carbonate baseline electrolytes, facilitating faster interfacial charge transfer. These solvation characteristics manifest in experimental observables such as the suppression of free-anion Raman bands or chemical-shift changes in nuclear magnetic resonance (NMR) spectra. Taken together, these criteria provide operational guidelines that complement the DN/*ε*/electrostatic potential (ESP) descriptors, offering a practical means to distinguish WSEs from both conventional dilute electrolytes and LHCE formulations. The following sections will expand on these key properties and discuss how Raman and NMR can be applied to characterize solvation structures in WSEs.

### 2.2. Key solvents and main properties

At the core of WSE design is the control of electrostatic interactions between metal cations and their surrounding molecular environment, as these forces fundamentally govern the architecture of the electrolyte solvation shell. From a physicochemical perspective, solvation is not merely a passive consequence of concentration or salt choice, but an active competition between ion–ion coulombic attractions and ion–dipole stabilization by solvent molecules.^29^ Ion–ion interactions, primarily between cations and anions, are dictated by charge density and separation distance, and tend to dominate when solvents are weakly polar or unable to screen electrostatics effectively. In contrast, ion–dipole interactions arise from local electrostatic alignment between the cation's positive charge and the negative end of a solvent dipole and are responsible for solvent coordination in conventional electrolytes.^30^

The nature and strength of electrostatic interactions in WSEs are governed by key solvent properties such as *ε*, DN, solvation energy, steric accessibility, and surface electrostatic potential.^31^ Together, these descriptors shape the electrostatic environment experienced by the cation, determining whether it is preferentially stabilized by solvent molecules or by anions. In doing so, they directly control the solvation structure, which in turn dictates desolvation energetics, interfacial reactivity, and ion transport behavior in WSEs.

Among the solvent-level properties that govern electrostatic interactions, the *ε* is perhaps the most global. It defines the extent to which the bulk medium can screen coulombic forces between charged species. In low-*ε* solvents, the electrostatic field around the cation remains strong and long-ranged, amplifying attraction to counter-anions and reducing the effectiveness of neutral solvent molecules at stabilizing the cation. By contrast, in high-*ε* environments, these fields are screened more effectively, allowing solvents to stabilize cations through dipolar interactions. Thus, in WSE design, solvents with *ε* typically below 10 are chosen to preserve ion–ion interactions and diminish solvent–cation electrostatic stabilization. Representative examples include cyclopentyl methyl ether (CPME, *ε* ≈ 4.8) and 1,1,2,2-tetrafluoroethyl ether (TFEE, *ε* ≈ 6.9), both of which exhibit limited dielectric screening and support anion-rich solvation structures. Conversely, high-*ε* solvents like ethylene carbonate (EC, *ε* ≈ 89.6) promote full salt dissociation and strong cation solvation, undesirable traits in WSEs.^29^ However, fluorination of an organic molecule normally reduces the *ε* of the molecule,^32,33^ thus partial fluorination of an organic solvent at selected locations and fluorination degree can tune the solvent properties to enable the formation of WSEs and achieve advanced battery performance.^34,35^

Complementing this bulk effect is the DN, which quantifies how strongly a solvent can donate electron density to a cation. This parameter captures the strength of ion–dipole interactions—the primary mode of cation–solvent binding. A high DN implies that the solvent can act as a strong Lewis base, anchoring the cation *via* localized negative charge. Solvents with low DN, by contrast, generate weaker ion–dipole interactions and are thus less effective at stabilizing the cation electrostatically. WSE-compatible solvents tend to exhibit DN values below ∼12. For example, dimethyl sulfoxide (DMSO, DN = 29.8) forms very strong cation–solvent complexes and is not suitable for WSEs, while DME (DN ≈ 20) remains borderline. In contrast, CPME (DN = 9.6) and TFEE (DN ≈ 11.2) strike the desired balance between weak solvation and sufficient salt compatibility.^36^

Interestingly, this interdependence between *ε* and DN was explored in detail by Wang *et al.* in their study on Na metal batteries.^37^ They introduced a weakly coordinating-intervention strategy and demonstrated that solvents exhibiting both low *ε* and low DN promote the formation of Na^+^–anion solvation structures, in contrast to the solvent-separated configurations typical of conventional LCEs. This synergistic reduction in polarity and donor strength effectively suppressed solvent–cation coordination and facilitated the incorporation of anions into the primary solvation shell. As a result, more robust and uniform SEI layer formed at the metal interface. Their findings highlight that tuning *ε* and DN can serve as a rational design strategy for manipulating solvation structure and optimizing interfacial stability in WSEs.

Solvation energy, often derived from density functional theory (DFT) or molecular dynamics (MD) calculations, offers a quantitative measure of the net electrostatic stabilization provided by a given solvent–cation pair. It reflects the depth of the potential energy well formed when a solvent binds to a cation. In WSEs, the goal is to maintain relatively shallow wells, typically in the range of −20 to −50 kcal mol^−1^. This ensures that the cation is not tightly held by the solvent and remains accessible for anion coordination. For instance, the Li^+^–DMSO interaction is exceptionally strong (≈−120 to −130 kcal mol^−1^) and hinders desolvation, while solvents like 1,3-dioxlane (DOL), 1,2-diethoxyethane (12DEE), and TFEE show binding energies within the WSE-compatible range, supporting weak solvation and favorable interfacial kinetics.^38^

While *ε*, DN, and solvation energy capture thermodynamic and dielectric aspects of electrostatic interaction, steric effects introduce a spatial constraint. Bulky or conformationally restricted solvents impose geometric limits on how closely their donor atoms can approach the cation. This limits the spatial overlap of their negative electrostatic field with the cation's positive charge, effectively reducing the strength of the ion–dipole interaction. Steric hindrance does not alter the *ε* or DN directly, but it weakens coordination by physically blocking optimal electrostatic alignment. Typical structural motifs include branched or fluorinated groups, such as those in TFEE, 1,1,2,2-tetrafluoroethyl-2,2,3,3-tetrafluoropropyl ether (TTE), or 1,1,2,2-tetrafluoroethyl methyl ether, or rigid ring systems like CPME.^39^

Finally, ESP maps, and specifically the negative ESP (ESP_min_), provide a molecular-level descriptor of how charge is distributed over the solvent's surface. ESP_min_ reflects the intensity of the most negative potential region on the molecule—typically where cation binding would occur. A strongly negative ESP_min_ indicates a region with high electron density and thus high electrostatic attraction for cations. In WSE design, moderate ESP_min_ values above −50 kcal mol^−1^ are desired to limit this attraction. For example, DMSO and EC exhibit highly negative ESP_min_ values (<−75 kcal mol^−1^), which correlate with their strong cation binding. In contrast, solvents like TFEE and CPME show moderate ESP_min_ values (≈−40 to −50 kcal mol^−1^), aligning with their weakly solvating behavior. While ESP maximum is more relevant to solvent–anion interactions, ESP_min_ remains the key predictor of how strongly a solvent is likely to stabilize a cation electrostatically.^36^

Together, these descriptors form a mechanistic toolkit for evaluating and designing WSEs. Their individual values are informative, but it is their combined effect on the electrostatic environment that ultimately determines whether a solvent will support or resist coordination. Importantly, WSE design is not about minimizing each descriptor independently; solvents that are too weakly interacting may fail to solvate the salt altogether or act merely as passive diluents, undermining their function as WSSs. The goal is not zero coordination, but deliberate suppression. In this sense, the design of WSEs is less about selecting “low” values across the board, and more about positioning the solvent in a regime where it consistently loses the coordination competition to the anion. This fine balance is what differentiates a functional WSS from an inert diluent.

**Fig. 2:**
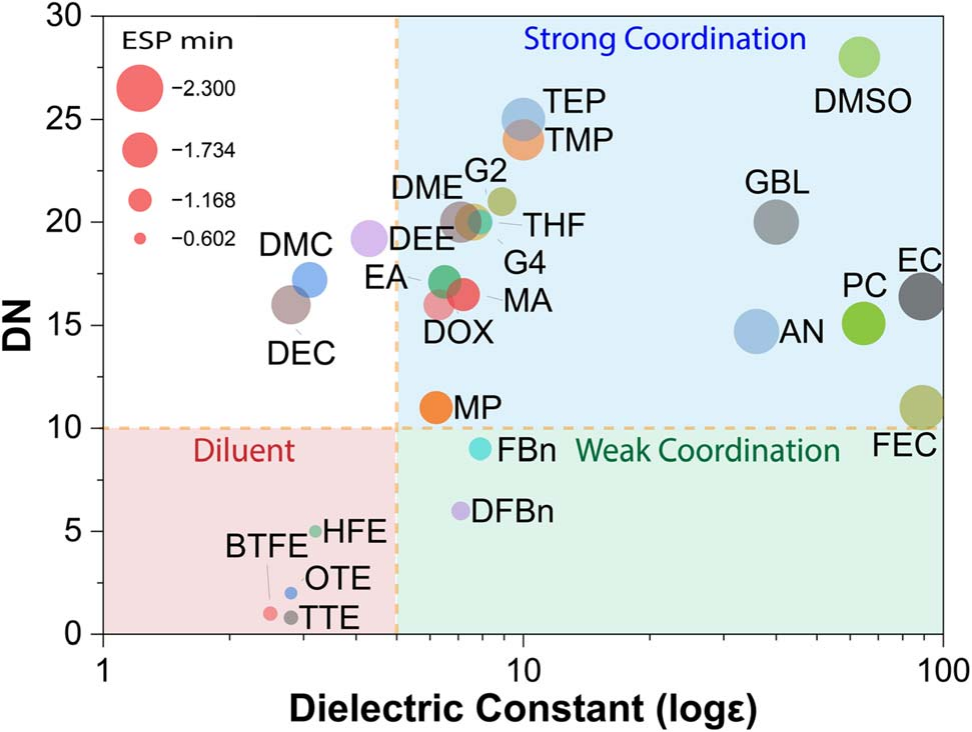
Diagram of donor number (DN), dielectric constant (*ε*) and negative electrostatic potential (ESP_min_) properties of different solvents. The diagram is plotted using the data reported in ref. 37, 40, 41.

As shown in Fig. 2 and Table 1, WSSs consistently occupy a distinct region of the *ε*–DN space, characterized by low *ε*, low DN, and moderate ESP_min_ value. This convergence of properties reflects a coordinated suppression of ion–dipole interactions, allowing anions to outcompete solvents in occupying the cation's primary solvation shell. Notably, solvents positioned near the boundary of this regime often exhibit transitional behavior, supporting limited cation coordination but requiring careful salt pairing or cosolvent design to maintain weak solvation. The spatial clustering observed underscores that WSE behavior is not dictated by a single parameter, but by a balanced electrostatic landscape in which the solvent consistently loses the coordination competition to the anion.

**Table 1:** Donor number (DN), dielectric constant (*ε*) and negative electrostatic potential (ESP_min_) properties of different solvents shown in Fig. 2

#	Solvent	Full name	ε	DN	ESP_min_ (eV)	Ref. #
1	FEC	Fluoroethylene carbonate	107	11	−2.2	37 and 40
2	EC	Ethylene carbonate	89.1	16.4	−2.3	37, 40 and 41
3	PC	Propylene carbonate	64.6	15.1	−2.1	37, 40 and 41
4	DMSO	Dimethyl sulfoxide	63.1	28	−2	41
5	GBL	γ-Butyrolactone	40	20	−2.2	37 and 40
6	AN	Acetonitrile	35.9	14.7	−2.2	40 and 41
7	TMP	Trimethyl phosphate	10	24	−2	40
8	TEP	Triethyl phosphate	10	25	−2.1	41
9	G2	Diethylene glycol dimethyl ether	8.9	21	−1.4	40
10	FBn	Fluorobenzene	7.9	9	−1.1	41
11	G4	Tetraethylene glycol dimethyl ether	7.9	20	−1.2	37 and 41
12	THF	Tetrahydrofuran	7.6	20	−1.8	37 and 41
13	MA	Methyl acetate	7.2	16.5	−1.6	40
14	DFBn	1,2-Difluorobenzene	7.1	6	−0.9	37 and 41
15	DME	1,2-Dimethoxyethane	7.1	20	−2	37 and 41
16	EA	Ethyl acetate	6.5	17.1	−1.6	41
17	DOX	1,4-Dioxane	6.3	16	−1.5	37
18	MP	Methyl propionate	6.2	11	−1.6	40
19	HFE	1,1,2,2-Tetrafluoroethyl-2,2,2-trifluoroethyl ether	3.2	5	−0.6	37 and 41
20	DMC	Dimethyl carbonate	3.1	17.2	−1.7	37 and 41
21	DEC	Diethyl carbonate	2.8	16	−1.9	37 and 40
22	TTE	1,1,2,2-Tetrafluoroethyl-2,2,3,3-tetrafluoropropyl ether	2.8	0.8	−0.7	40
23	OTE	Octafluoropentyl tetrafluoroethyl ether	2.8	2	−0.6	37, 40 and 41
24	BTFE	Bis(2,2,2-trifluoroethyl) ether	2.5	1	−0.7	37, 40 and 41
25	DEE	Diethyl ether	4.3	19.2	−1.8	41

While solvation descriptors such as DN, *ε*, and electrostatic potential provide the framework for identifying weakly solvating solvents, practical electrolyte evaluation ultimately depends on whether sufficient ionic conductivity can be maintained under realistic conditions. Conventional carbonate electrolytes still represent the upper benchmark (>7 mS cm^−1^ at room temperature), but their strong cation–solvent coordination imposes large desolvation penalties and interfacial polarization. For this reason, the comparison between LHCEs and WSEs is more instructive. LHCEs achieve interfacial stability by forcing anion participation into the solvation shell, but this comes at the cost of transport.^28^ The reliance on inert diluents and clustered ionic aggregates suppresses conductivity and lowers the effective cation transference number. For example, variant LHCEs can sustain only ∼1 mS cm^−1^ at −40 °C^28^ and even quasi-LHCEs require elevated salt contents and fluorinated cosolvents to recover ∼9 mS cm^−1^ at room temperature.^42^ These formulations highlight both the potential and limitations of the LHCE strategy: while oxidative stability and interfacial robustness are improved, transport remains compromised or achievable only through costly and complex electrolyte mixtures.

WSEs approach the same objective by a different mechanism. By suppressing strong cation–solvent binding at conventional salt concentrations, WSEs promote anion-rich coordination without resorting to excessive salt loading or non-participating diluents. As a result, they maintain moderate but stable ionic conductivities in the 2–5 mS cm^−1^ range, values sufficient for practical operation. In aqueous zinc systems, hydrated eutectic formulations achieve ∼3–5 mS cm^−1 43^. Non-aqueous eutectics based on fluorinated amides reach ∼3 mS cm^−1 44^. Na electrolytes using weak ethers report ∼5 mS cm^−1^ at 25 °C and still retain 1.8 mS cm^−1^ at −20 °C,^45^ demonstrating thermal robustness. Li WSEs built from weakly coordinating ethers exhibit somewhat lower conductivities (∼1–2 mS cm^−1^),^46^ but combine this with high Li^+^ transference numbers and lower desolvation barriers, yielding superior rate capability.

The key distinction is not simply the magnitude of conductivity but the pathway by which it is achieved. LHCEs often reach comparable values only through extreme formulations that increase viscosity, reduce mobility, or raise cost. In contrast, WSEs sustain conductivity under simpler chemistries while simultaneously enhancing interfacial kinetics. The combination of moderate *σ*, high *t*^+^, and reduced desolvation energy provides a transport environment that is inherently more balanced. This balance explains why WSEs frequently outperform LHCEs in rate performance and wide-temperature operation, despite working with bulk conductivities that are not maximized. In this sense, the weakly solvating strategy provides a more direct and scalable route to coupling solvation chemistry with effective ion transport.

### 2.3. Characterizing solvation structures in WSEs

**Fig. 3:**
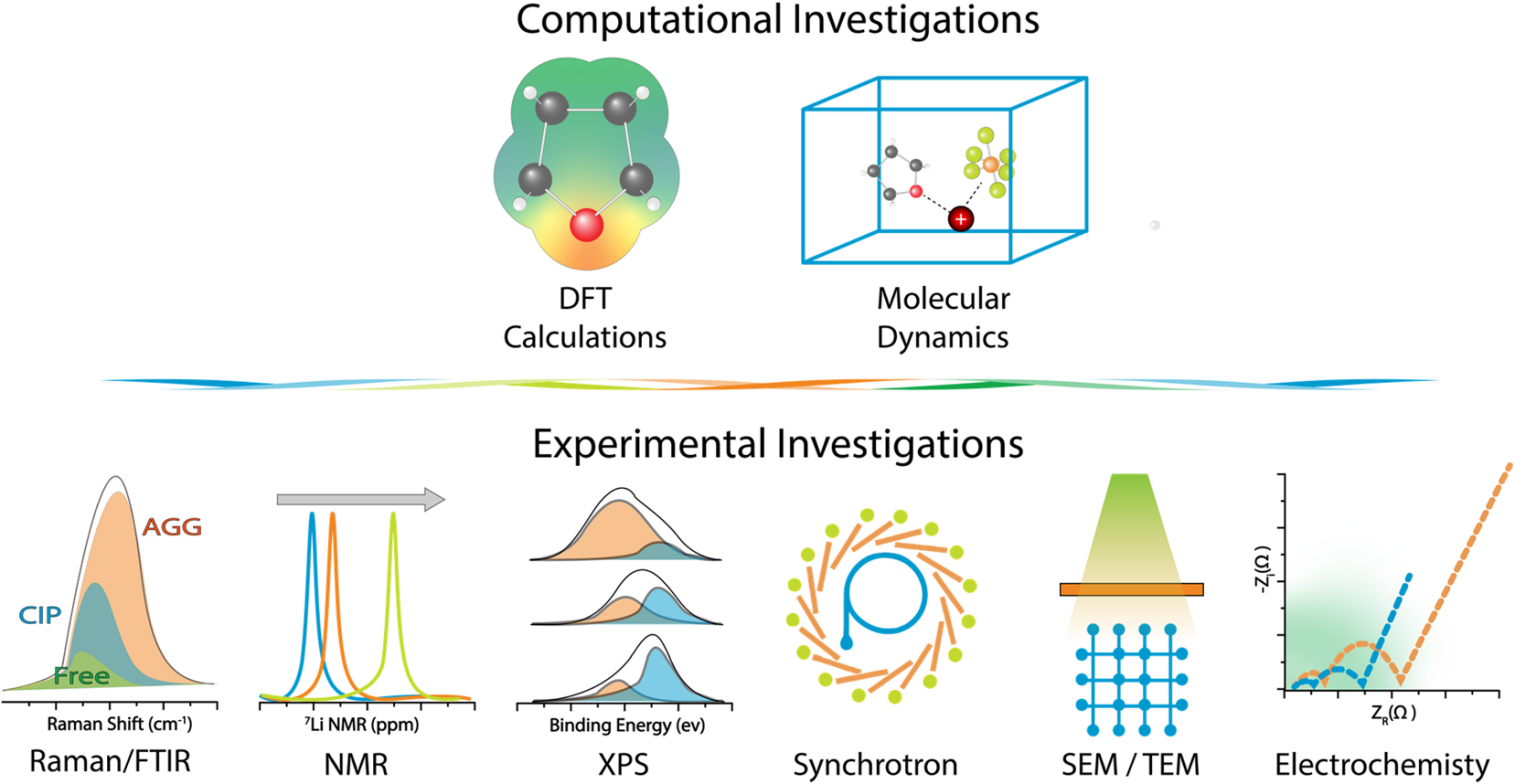
Computational and experimental approaches to investigate WSEs.

Understanding how metal ions interact with their immediate molecular environment is essential for designing WSEs. Characterizing this altered solvation regime demands an integrated approach, combining predictive theoretical modeling with experimental techniques capable of resolving both structural and electronic features across atomic to mesoscale dimensions. This section presents a unified analysis of the methods currently used to probe WSE solvation environments, starting with simulation-based descriptors and proceeding to spectroscopic and imaging tools used for experimental validation. Fig. 3 exhibits the different computational and experiment approaches to investigate WSEs.

Computational investigations of solvation in WSEs rely heavily on the complementary use of DFT calculations and MD simulations. DFT is often used to quantify the thermodynamic and electronic landscape of cation–solvent–anion interactions through descriptors such as desolvation energy, binding energy, and frontier orbital alignment. These parameters help elucidate which species in solution are most likely to undergo reduction or oxidation at the electrode surface. For instance, comparing Na^+^ solvation in 1,2-dimethoxyethane (DME) and 2-methyltetrahydrofuran (MeTHF) has shown that a lower desolvation energy in MeTHF correlates with a faster Na^+^ release from the solvation shell—an essential condition for high-rate performance.^45^ The same principles were applied when weakly coordinating ether solvents like 1-[(2-methoxyethoxy)methyl]-1-methylpyrrolidinium hexafluorophosphate (EOP) were introduced to replace conventional ethers. Here, DFT calculations indicated a shift in the lowest unoccupied molecular orbital (LUMO) localization from the solvent to the PF_6_^−^ anion, suggesting a reorganization of decomposition pathways toward anion-dominated interphase formation.^47^ Importantly, these shifts were not only electronic but also structural, as verified by MD simulations that revealed a PF_6_^−^-rich solvation environment, reducing Na^+^ desolvation energy by nearly 60 kcal mol^−1^ and enabling rapid transport coupled with robust SEI formation.

While DFT provides a static picture of the most stable configurations, MD simulations capture the dynamic evolution of solvation structure in response to changing concentration or composition. Classical MD (CMD) and *ab initio* MD (AIMD) techniques yield radial distribution function, coordination number, and solvation shell lifetime that quantify the temporal and spatial arrangement of species around the cation. In LiFSI-12DEE systems, such simulations revealed a concentration-dependent shift from solvent-separated to anion-coordinated environments.^25^ At higher salt concentrations, Li^+^ lost solvent oxygen (O) neighbors and increasingly coordinated with FSI^−^, forming CIPs and AGGs. These configurations were then extracted and subjected to DFT calculations, which confirmed the orbital localization on FSI^−^ in high-concentration systems, signaling the emergence of WSE-like behavior. Similar MD-based analysis in aqueous Zn^2+^ systems has shown that steric hindrance from ligand methylation disrupts Zn–ligand interactions, allowing more water coordination and easier desolvation. Collectively, these simulations go beyond descriptive statistics: they reveal how solvation geometry, energetics, and electronic structure evolve under specific molecular constraints, enabling predictive control over the electrolyte's interfacial chemistry.^43^

These computational insights must be validated under real conditions, and experimental tools offer critical access to solvation structure, interphase composition, and morphological evolution. Raman spectroscopy is especially powerful in this regard, as it sensitively tracks shifts in vibrational modes associated with ion coordination.^48,49^ In systems like LiFSI-12DEE, deconvolution of FSI^−^ S–N–S symmetric stretches revealed the progressive transformation of solvation species from free anions and SSIPs to CIPs and AGGs. As salt concentration increased, peaks associated with free FSI^−^ disappeared, replaced by dominant AGG signals, which comprised over 80% of the total at 4.5 M.^25^ These observations, consistent with MD predictions, directly confirm that WSEs suppress solvent–cation interactions and restructure the solvation environment toward compact, ionically associated states that favor interfacial stability.

Fourier transform infrared (FTIR) spectroscopy complements Raman by probing bond vibrations and hydrogen bonding dynamics, which are sensitive to solvent–ion and solvent–solvent interactions. In aqueous Zn(OTf)_2_ systems modified with dioxane, FTIR revealed redshifts in CH_2_ bending vibrations and weakening of O–H stretching bands, clear signs of disrupted water coordination and stronger anion participation in the solvation shell.^50^ Analogous trends have been observed in organic systems, where acetonitrile (AN) and tetrahydrofuran (THF) disrupted conventional solvation structures and promoted tighter ion clusters. These results provide not only qualitative evidence of reduced solvent involvement but also a molecular fingerprint of solvation structure evolution in WSEs.^51,52^

NMR spectroscopy extends this insight into the electronic environment of specific nuclei. ^7^Li NMR, for instance, reveals shifts in chemical environments as fluorinated solvents reduce Li^+^–solvent interactions and promote Li^+^–anion pairing. This trend was further quantified using diffusion-ordered spectroscopy (DOSY)-NMR, which measured reduced diffusion coefficients and decreasing solvent coordination numbers with increasing fluorination, providing clear, quantitative markers of solvation shell compaction.^53^^11^B and ^19^F NMR techniques applied to BF_4_^−^ and DFOB^−^ systems have shown similar shifts and peak broadenings in WSEs, indicating increased anion participation around Li^+^.^54^ When interpreted alongside Raman and FTIR, these NMR-based observations provide a highly resolved picture of how solvation structure evolves with solvent design and salt chemistry.

While bulk-phase solvation analysis provides crucial mechanistic insight, understanding the performance of WSEs requires probing the electrode–electrolyte interface where decomposition and interphase formation occur. X-ray photoelectron spectroscopy (XPS) offers depth-sensitive analysis of SEI and CEI chemical compositions. In WSEs employing fluorinated additives like 2,3-difluoro-5-(trifluoromethyl)pyridine (DP), XPS revealed a layered SEI architecture dominated by LiF and Li_3_N.^46^ Organic species such as RCOOLi and C–O fragments were minimal and decreased with etching depth, confirming that solvent decomposition was suppressed and interphase formation was governed by anion or additive-derived products. Such inorganic-rich SEIs are key to long-term stability in metal batteries and are one of the defining advantages of WSE architectures.

To understand how these interphases evolve in space, synchrotron-based imaging and scattering techniques provide nanoscale and 3D perspectives. Synchrotron X-ray computed tomography (CT) has visualized changes in void formation and electrode morphology upon cycling in WSEs. In SiO anodes, switching to a WSE containing succinonitrile (SN) reduced porosity from 10.6% to 3.9%, demonstrating improved mechanical stability linked directly to electrolyte design.^55^ At the molecular scale, wide-angle X-ray scattering (WAXS) and small-angle X-ray scattering (SAXS) techniques have been used to track ion clustering and nanoscale ordering. High-entropy electrolytes studied using these techniques showed that increasing solvent diversity leads to smaller, more mobile ion clusters, a result consistent with WSE theory and directly linked to improved interfacial uniformity.^56^

Finally, imaging tools such as scanning electron microscopy (SEM), transmission electron microscopy (TEM), and cryo-TEM reveal the structural signatures of WSE-derived interphases. SEM captures surface morphology and dendrite suppression; TEM provides crystallographic and phase distribution data; and cryo-TEM, with its ability to preserve fragile and beam-sensitive domains, has emerged as an essential method for visualizing amorphous or nanocrystalline SEI phases.^57,58^ When coupled with spatially resolved spectroscopy (*e.g.*, energy dispersive X-ray spectroscopy (EDS), electron energy loss spectroscopy (EELS)), these imaging methods confirm the spatial distribution of inorganic and organic components in WSE-derived interphases. Collectively, they complete the mechanistic picture, linking solvation structure to interfacial evolution, morphological stability, and ultimately to electrochemical performances.

## 3. WSEs for alkali metal batteries

### 3.1. WSEs for Li metal batteries

Li metal systems have been the primary testbed for WSE strategies. Their wide voltage requirements, reactive interfaces, and compatibility with high-energy cathodes make them an ideal environment to evaluate how solvation structure governs interfacial chemistry. In addition, Li metal batteries offer a rich set of materials and characterization tools to study how electrolyte design affects cycling, CE, and rate performance. Three major strategies have emerged across literature: tuning solvent structure to intrinsically reduce coordination strength, combining solvents with differentiated functions, and modifying anion chemistry to outcompete solvent coordination. This section outlines how each approach manifests in Li systems, and what interfacial and electrochemical performance improvements it enables.

**Fig. 4:**
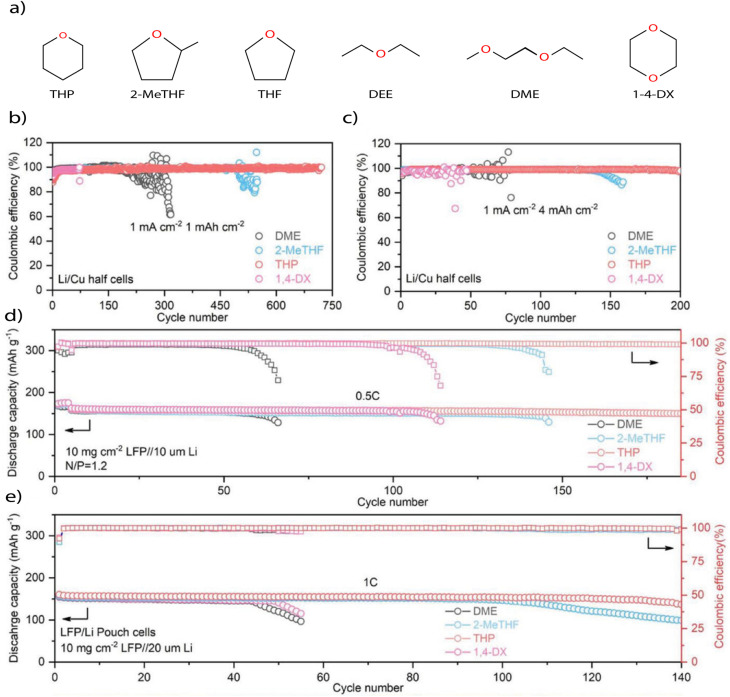
(a) Chemical structures of classic ether solvents; (b) and (c) CE of Li‖Cu half cells at (b) 1 mA cm^−2^, 1 mAh cm^−2^, (c) 1 mA cm^−2^, 4 mAh cm^−2^; and (d) and (e) cycling performance test of LFP‖Li in (d) coin cells and (e) pouch cells. Reproduced with permission.^59^ copyright 2023, Wiley.

One of the clearest WSE strategies is to employ solvents that inherently bind Li^+^ weakly. This can arise from low DN, low *ε*, low ESP at the coordinating site (ESP_min_), or steric hindrance around the O atom. Solvents with these features reduce the population of SSIPs, and favor CIPs or AGGs, where anions dominate the primary solvation shell. Among different ether solvents, cyclic ethers like tetrahydropyran (THP) exhibit low solvation strength due to their symmetric geometry and reduced O electron density, leading to reduced Li^+^–O binding energy. These electrolytes generate SEIs rich in LiF, suppress dendrites, and enable high CE (99%) in Li‖Cu cells (Fig. 4b and c) and LFP‖Li coin cells and pouch cells (Fig. 4d and e) over hundreds of cycles.^59^ Similarly, siloxane solvents, such as dimethoxydimethylsilane (DDS) and tetramethyl orthosilicate (TMOS), weaken Li^+^ binding due to the larger atomic radius of silicon (Si), which lowers the orbital overlap and bond strength in Si–O–Li coordination. This enables anion-dominant solvation without the need for high salt concentrations, supporting stable plating and high-voltage cycling up to 4.5 V.^60^

A more chemically fine-tuned system combines two fluorinated esters, ethyl difluoroacetate (2F) and ethyl trifluoroacetate (3F), at a 3 : 7 volume ratio in a 0.5 M LiPF_6_ electrolyte. Despite the low salt concentration, 2F offers moderate coordination ability with Li^+^, while 3F acts as a non-coordinating diluent that enhances PF_6_^−^ entry into the solvation shell. The resulting solvation structure lowers Li^+^ desolvation energy (from 17.5 to 11.2 kJ mol^−1^), promotes formation of a LiF-rich CEI, and improves interfacial kinetics. Electrochemically, the system exhibits faster Li^+^ diffusion, lower polarization, and delivers high-rate performance (130 mAh g^−1^ at 4 C) in LFP‖Li cells.^61^

**Fig. 5:**
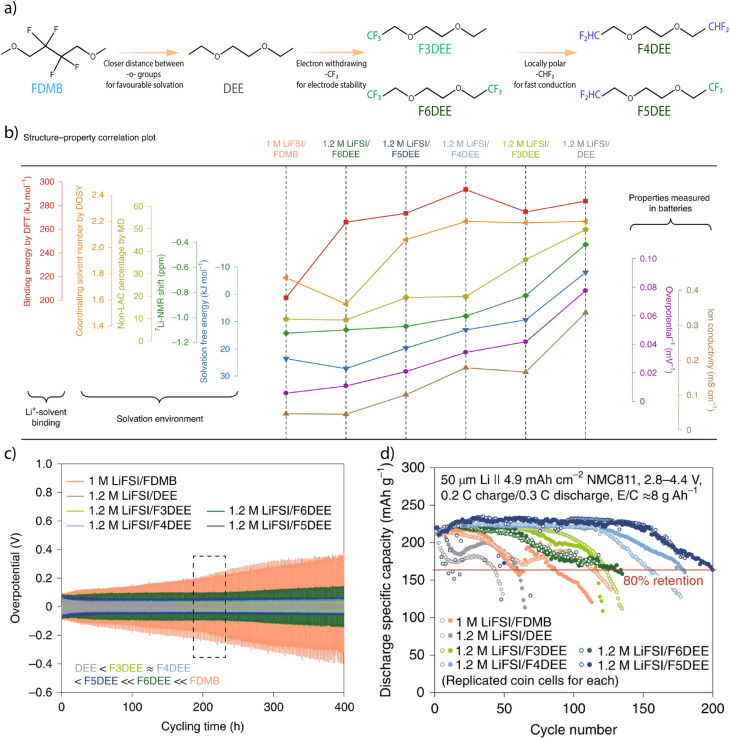
(a) Step-by-step design principles of the fluorinated-12DEE solvent family, and (b) structure–property relationship plot of Li^+^–solvent binding, solvation environments and properties measured in batteries. (c) Cycling performance of Li‖Li symmetric cells. (d) Long-cycling performance of thin Li‖high-loading-NMC811 coin cells. Conditions: 50 μm thick Li, 4.9 mAh cm^−2^ NMC811, 2.8–4.4 V, 0.2 C charge and 0.3 C discharge, and electrolyte-to-capacity ratio (E/C) = 8 g (Ah)^−1^. Reproduced with permission.^34^ Copyright 2022, Nature.

Through a series of studies, Bao and coworkers have demonstrated that partial fluorination of ether solvents provides a viable molecular pathway to achieve WSEs.^34^ By incorporating asymmetric –CHF_2_ groups or tuning the fluorination degree on 12DEE backbones (Fig. 5a), these systems reduce the donor strength of the solvent, consequently lower the dielectric constant due to diminished polarity and polarizability and shift the solvation structure toward CIPs and anion-rich AGGs. Solvents with different fluorination states exhibit suppressed Li^+^–solvent coordination and enhanced anion participation, yielding solvation environments consistent with WSE behavior at a salt concentration as low as 0.5 M. Fig. 5b represents the relationship between Li^+^ binding and solvation environment of electrolytes using different partially fluorinated solvents. Electrochemical performance was further assessed using symmetric Li‖Li cells (Fig. 5c), where the overpotentials mirrored the inverse trend of ionic conductivities shown in Fig. 5b. The fully fluorinated electrolyte exhibited significantly higher overpotential due to its low ionic conductivity and poor Li^+^ mobility. In contrast, the partially fluorinated solvents achieved a favorable balance between ionic conductivity and weak Li^+^ coordination, resulting in lower and more stable overpotentials throughout cycling. Furthermore, Li‖NMC 811 cells were cycled, confirming the trend of superior capacity retention of partially fluorinated electrolytes maintaining 80% capacity after 200 cycles compared with the baseline electrolyte. These results illustrate how rational modulation of electron density and molecular dipole enables the design of WSEs without requiring complex multicomponent formulations or extreme salt loading.^35^

While solvent design can weaken Li^+^ coordination, another strategy is to directly enhance the competitiveness of the anion. This can be done by increasing its DN, charge localization, or steric profile to favor direct coordination with Li^+^. For example, replacing a –CF_3_ group in TFSI^−^ with a –CH_3_ to form MTFSI^−^ increases the basicity and nucleophilicity of the anion. This allows it to displace the highly solvating binary solvents (EC/diethyl carbonate (DEC)) from the primary solvation shell, shifting the solvation structure toward anion-rich coordination. The resulting SEI is composed of robust inorganic species such as Li_2_S and Li_3_N, leading to >98.9% CE and >150 stable cycles in high-loading pouch cells.^62^

**Fig. 6:**
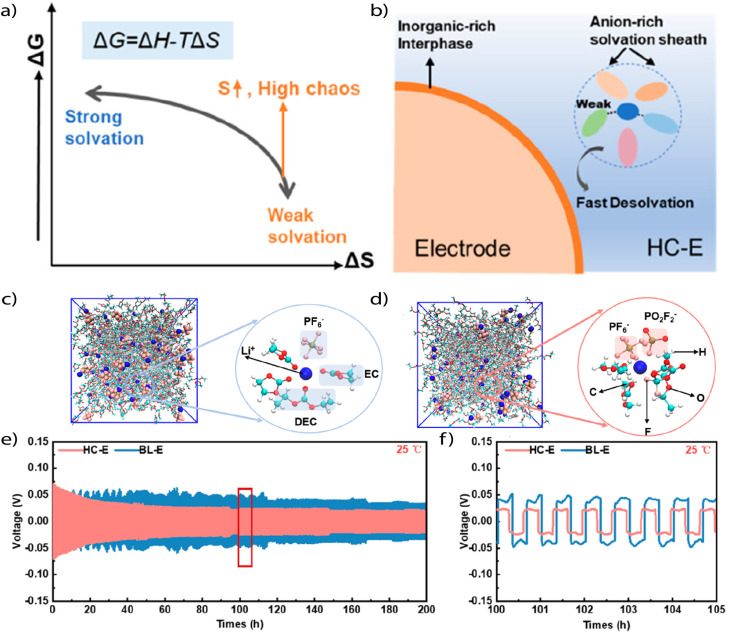
(a) Design concept of improving chaos, (b) optimization mechanism of the high chaos electrolytes. (c) and (d) Snapshots of molecular dynamic simulation for (c) high chaos electrolyte and (d) baseline electrolyte. (e) and (f) Cycling stability of Li‖Li symmetric cells obtained at 0.1 mA cm^−2^. Reproduced with permission.^63^ Copyright 2023, American Chemical Society.

More recently, high-entropy electrolytes have been explored as a distinct strategy to stabilize Li metal interfaces and promote anion-rich solvation structures even at conventional salt concentrations. This approach introduces multiple types of anions into the system, generating a heterogeneous solvation environment with small, diverse solvation clusters (Fig. 6a and b). The resulting configuration facilitates ion diffusivity and promotes high ionic conductivity by reducing structural order and enhancing the statistical likelihood of anion coordination. Although originally proposed as a separate electrolyte category, these systems inherently achieve weak solvation characteristics and thus fall within the design space of WSEs.^56^ For instance, a “high chaos” electrolyte composed of LiPF_6_, LiDFOB, and LiPO_2_F_2_ in ethyl methyl carbonate (EMC) and dimethyl carbonate (DMC) creates a large number of accessible solvation configurations. This configurational entropy statistically favors anion inclusion in the solvation shell, promoting formation of CIPs and AGGs without requiring high salt concentration (Fig. 6c and d). These electrolytes suppressed solvent decomposition, stabilized SEI and CEI formation, and enabled high voltage cycling up to 4.7 V across −30 °C to +45 °C. In addition, the cycling performance in a symmetrical Li‖Li cell showed a low and stable overpotential over 200 h for the high chaos electrolytes compared to the typical counterpart.^63^

It is worth noting, however, that high-entropy or “chaotic” electrolytes are not automatically equivalent to WSEs. Their ability to promote anion participation often arises from configurational entropy and statistical mixing rather than intrinsically weak solvent–cation interactions. In some formulations, this entropy-driven disorder indeed mimics WSE-like solvation by reducing the effective coordination of strongly binding solvents. In others, particularly where high-DN or high-*ε* solvents remain dominant in the first solvation shell, the outcome diverges from the weakly solvating regime. High-entropy electrolytes should therefore be viewed as a complementary design space, where they can converge with WSE principles when solvent–cation binding is sufficiently suppressed, but remain conceptually distinct when solvation is still governed by strongly coordinating solvents.

**Fig. 7:**
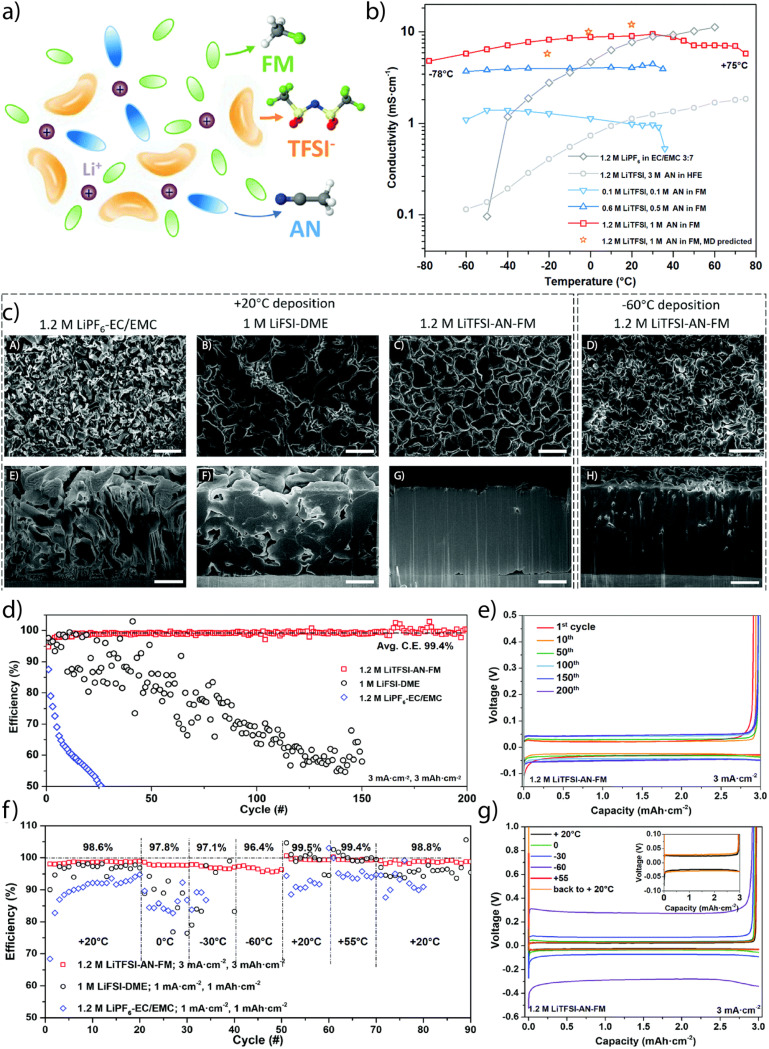
(a) Schematic illustration of the solvation structure of liquefied gas electrolyte 1.2 M LiTFSI, 1 M AN in FM. (b) Temperature dependence of ionic conductivity of liquefied gas electrolytes with different salt and cosolvent concentrations from experiments and the MD simulations predictions for 1.2 M LiTFSI, 1 M AN in FM. (c) Cryo-FIB characterization of the morphologies of electrochemically deposited Li (A–D) top-view SEM images (A–C, scale bar 10 μm; (D) scale bar 5 μm), (E–H) cross-sectional SEM images (scale bar 4 μm) of deposited Li. The Li metal was plated in 1.2 M LiPF_6_-EC/EMC (A and E), 1 M LiFSI-DME (B and F), and 1.2 M LiTFSI-AN-FM (C and G), at a current density of 0.5 mA cm^−2^ with a capacity of 3 mAh cm^−2^ at room temperature. Li metal in (D and H) was deposited at −60 °C in the same liquified gas electrolyte, current and capacity. (d) CE of Li metal plating/stripping over 200 cycles in various electrolytes, and (e) voltage profiles for the cell using liquefied gas electrolyte. (f) CE of Li metal plating and stripping at various temperatures, and (g) voltage profiles for the cell using liquefied gas electrolyte. Reproduced with permission.^64,65^ Copyright 2020, Royal Society of Chemistry.

Liquefied gas electrolytes (LGEs) offer an alternative WSE strategy by leveraging solvents with inherently low solvation strength, such as liquefied fluoromethane (FM). Their low DN, small molecular size, and low polarizability limit solvent–Li^+^ interaction, favoring solvation structures rich in anions and tightly bound cosolvents (Fig. 7a). When paired with sub-stoichiometric amounts of AN or THF, the resulting electrolyte shows high Li^+^ transference number (∼0.9), fast ion transport (Fig. 7b), and dense Li deposition even at −60 °C (Fig. 7c). These systems support CEs >99.4% and stable cycling across −60 to +55 °C (Fig. 7d–g). Although originally developed for wide-temperature operation, LGEs fulfill the structural and interfacial criteria of WSEs and illustrate how phase-state engineering can enable weak solvation behavior.^64,65^ It should be noted that heavy, pressurized containers are required to keep the gas components in liquefied phase in the electrolytes, which largely reduces the specific energy of the batteries.

Weakly solvating design also provides a pathway to extend electrolyte stability toward elevated temperatures, not only cryogenic regimes. Cycloalkyl ethers such as CPME exemplify this principle, offering a remarkably broad liquid range from −140 to +106 °C while maintaining weak Li^+^ coordination.^66^ When paired with fluorinated cosolvents, CPME-based electrolytes deliver stable cycling of practical Li-ion pouch cells from −60 °C up to >50 °C, demonstrating that weak solvation can unify low-temperature desolvation kinetics with high-temperature stability. Similarly, partially fluorinated ether systems configured as partially and weakly solvating electrolytes (PWSEs) stabilize Li‖LiCoO_2_ full cells across −20 to +60 °C by combining anion-rich solvation with robust interphase chemistry.^26^ Such capability is especially relevant for applications where devices must tolerate both freezing and elevated ambient conditions, ranging from electric vehicles in cold or hot climates to grid-scale storage and aerospace systems, underscoring the practical importance of solvation-centric design in real-world deployment.

### 3.2. WSEs for Na metal batteries

While Li metal batteries have required several generations of electrolyte engineering to reach high CE (≥99.5%), progressing from LCEs, HCEs, LHCEs to WSEs, the path has been notably more straightforward for Na metal systems. It is not an exaggeration to state that 99.5% CE has been achieved in Na metal batteries merely by identifying compatible solvents and salts, without necessitating extensive restructuring of the solvation environment.^67,68^ In fact, a recent work has demonstrated that Na metal full cells employing 1 M NaPF_6_ in DME and a controlled Na inventory can sustain an average CE of 99.91% over 500 cycles at 2 C rate, relying only on a simple ether-based electrolyte and modest stack pressure. This level of reversibility is obtained without resorting to highly engineered solvation chemistries, underscoring the inherently favorable Na–electrolyte interfacial compatibility in well-chosen systems. Such results highlight a fundamental divergence from Li, where comparable efficiencies typically demand complex solvation structure manipulation.^69^ Then the discrepancy raises a fundamental and underexplored question: Why is Li metal intrinsically harder to stabilize than Na metal? Surprisingly, the literature offers little mechanistic insight into this contrast. Few studies address it directly, and fewer still provide a unified framework. However, by deliberately suppressing solvent–cation coordination and enabling anion-rich solvation, WSEs may offer a lens through which this difference can be rationalized.

**Fig. 8:**
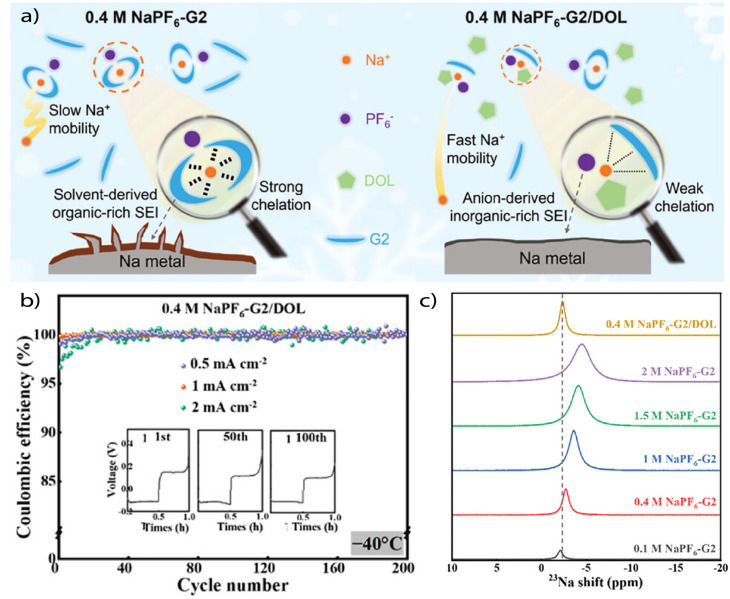
(a) Schematic illustration of the mechanism of improved low-temperature Na reversibility by the DOL-diluted electrolyte. (b) Cycling stability of Na‖Al cells at different current densities in 0.4 M NaPF_6_-G2/DOL under −40 °C. The insets show the corresponding voltage profiles at the 1st, 50th, and 100th plating/stripping cycles at 2 mA cm^−2^. (c) ^23^Na NMR spectra of the 0.4 M NaPF_6_-G2/DOL electrolyte and NaPF_6_-G2 electrolytes with different concentrations. Reproduced with permission.^70^ Copyright 2024, Wiley.

At the root of the divergence lies ion–solvent coordination thermodynamics, which are governed primarily by ionic charge density. Li^+^, being smaller (0.76 Å) and more charge-dense than Na^+^ (1.02 Å), exhibits stronger electrostatic interactions with Lewis basic donor sites. This leads to higher binding energies in typical solvents, particularly in O-coordinating species like glymes and carbonates. Consequently, Li salts tend to form tight, solvent-dominated solvation shells, requiring elevated energy input for desolvation. This tight binding also directs solvent decomposition pathways at the interface, leading to organic-rich SEIs and low reversibility unless desolvation is artificially facilitated. By contrast, Na^+^ exhibits weaker solvent coordination due to its larger ionic radius and lower charge density. This fundamental difference has several mechanistic consequences that directly impact interfacial stability and reversibility. First, the reduced electrostatic interaction between Na^+^ and coordinating solvent molecules lowers the desolvation energy barrier, facilitating faster cation reduction and more uniform metal deposition (Fig. 8a). This effect is particularly evident in systems such as the DOL-diglyme electrolyte studied by Hu *et al.*, where weakened Na^+^ solvation enabled CE values exceeding 99.9% even at −40 °C in Na‖aluminum (Al) cells (Fig. 8b).^70^ Second, the diminished solvent coordination at moderate salt concentrations allows for increased anion participation in the primary solvation shell. As a result, the interfacial decomposition is shifted away from solvent-dominated pathways and toward salt-derived species. This promotes the spontaneous formation of SEIs that are richer in inorganic components such as NaF, Na_3_PO_4_, and Na_2_CO_3_. These inorganic or mixed-type SEIs exhibit superior mechanical strength, higher ionic conductivity, and greater chemical uniformity compared to their organic-rich counterparts. Overall, this mechanistic contrast helps explain why Na metal attains very high efficiencies with comparatively simple ether electrolytes, whereas Li demands extensive solvation structure engineering to reach similar levels of reversibility. The inherently weaker Na^+^–solvent interactions lower desolvation barriers and guide interphase formation toward more inorganic products, providing a built-in advantage over Li. Even so, weakly solvating designs remain relevant for Na systems, particularly in enabling compatibility with high-voltage cathodes, suppressing parasitic reactions under practical cycling conditions, and extending stability across wide temperature ranges. In this way, Na not only illustrates how weak solvation principles manifest in a more forgiving alkali metal but also serves as a reference point for understanding electrolyte design across different chemistries.

Therefore, the physicochemical landscape of Na^+^ inherently promotes conditions that must be carefully engineered in Li^+^ systems. The weak solvation strength of Na ions effectively mimics WSE behavior even in relatively conventional electrolyte formulations. Importantly, this implies that solvation engineering is less of a prerequisite and more of an optimization strategy in Na metal batteries. For example, weakly solvating cosolvents like DOL, THF, or fluorinated ethers can still tune desolvation and enhance SEI uniformity, but high CE can often be accessed even in standard glyme-based systems. This has been observed across multiple studies, including Tanwar *et al.*, where stable Na plating/stripping on bare Al was achieved over hundreds of hours using tetraglyme-based electrolytes.^71^ Finally, the ability to achieve 99.9% CE at high current density and low temperature is a strong indicator of fast interfacial kinetics in Na systems. This is not solely a consequence of ionic conductivity but rather of reduced desolvation penalties and more favorable interphase formation.

**Fig. 9:**
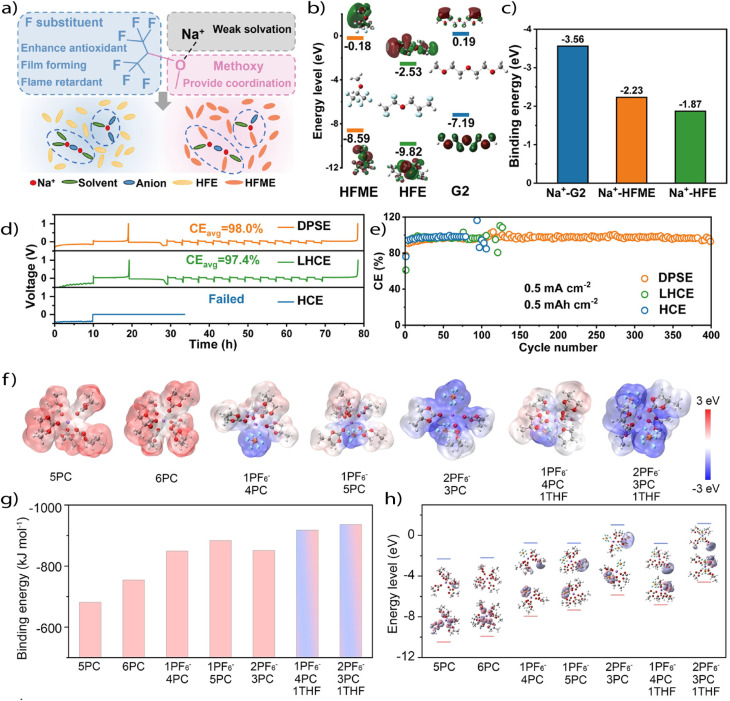
(a) Schematic representation of the solvation structure using HFME as a weakly coordinating diluent. (b) HOMO and LUMO energy levels of HFME, HFE, and G2. (c) Binding energies of G2, HFME, and HFE with Na^+^. (d) CE by the standard Aurbach method and (e) traditional CE of Na‖Cu cells in weakly coordinating electrolyte. Reproduced with permission^72^ Copyright 2024, Wiley. (f) Electrostatic potential density distribution, (b) binding energy, and (c) HOMO/LUMO energy level of representative solvation configurations. Reproduced with permission.^76^ Copyright 2024, Wiley.

Despite Na's inherent coordination weakness relative to Li, researchers have increasingly turned to WSEs to extend the operational envelope of Na metal batteries, particularly under high-voltage, high-rate, or low-temperature conditions. A major line of work has focused on reducing the strength of Na^+^–solvent interactions by selecting or introducing solvents that are intrinsically poor donors. These include weakly coordinating ethers such as THF, MeTHF, and fluorinated co-solvents that either sterically hinder coordination or withdraw electron density from donor atoms. For instance, fluorinated ether diluents such as hexafluoroisopropyl methyl ether (HFME) were shown to directly participate in the solvation shell while weakening the Na^+^–diglyme interaction (Fig. 9a–c), leading to lower desolvation energy and more robust interphase formation even under high-rate cycling,^72^ reaching high CE values in Na‖Cu cells (Fig. 9d and e). Further, a more aggressive strategy involves using charged or polarizing solvent molecules. Wu *et al.* employed an ionized ether (1-((2-methoxyethoxy) methyl)-1-methylpyrrolidinium hexafluorophosphate, EOP), which interacts with carbonate solvents to polarize the solvation structure and reduce solvent–cation binding. This electrolyte exhibited enhanced interfacial Na^+^ transport, high CE (>99.7%) even at a high rate (5 C) and voltage (2.0 to 4.5 V *vs.* Na/Na^+^), and excellent safety due to its nonflammability.^47^

Beyond modifying solvents, several studies have focused on strengthening anion coordination as the dominant influence on solvation and interfacial chemistry. Na systems are particularly well-suited for this, as their lower charge density allows anions to more readily enter the primary solvation shell at moderate salt concentrations. One approach uses high lattice energy salts like NaBF_4_ to enforce anion-dominated solvation, even in solvents that would not be considered weakly solvating for Li^+^. For example, Zhao *et al.* demonstrated that NaBF_4_ can reshape the solvation structure in DME/TEGDME (tetraethylene glycol dimethyl ether) mixtures, to generate a CIP/AGG-dominated coordination environment. Despite their moderate donor strength, the strong Na^+^–BF_4_^−^ interaction suppresses solvent coordination, driving the formation of anion-rich SEI. This architecture supports 5000-cycle stability at 20 C and 4.5 V in Na‖Na_3_V_2_(PO_4_)_2_F_3_ cells.^73^

Similarly, tuning the enthalpy of coordination through solvent chemistry, such as using fluorinated esters like fluoroethylene carbonate (FEC), has been used to shift coordination equilibrium toward PF_6_^−^, generating stable interphases and high CE at both the anode and cathode interfaces.^74^ A distinct variant of this approach is dual modulation of both anion and cation coordination, as shown in the work of using ethoxy(pentafluoro)cyclotriphosphazene (PFPN) as a co-solvent by Wang *et al.*,^75^ where PFPN simultaneously reduces Na^+^–PC and ClO_4_^−^–PC interactions, enabling a wider electrochemical window (4.84 V) and stable high-voltage cycling at low salt concentrations.

While these approaches center on solvation structure, others aim to recover bulk transport properties that are often compromised in WSEs. Mixed-solvent architecture provides a path to maintain weak solvation behavior while improving ionic conductivity and wettability. The pairing of THF with PC, for example, was shown to generate anion-enriched solvation environments with enhanced ionic conductivity due to entropy-driven solvent diversity (Fig. 9f–h). This allowed stable full-cell cycling with high mass loading and >80% capacity retention over 100 cycles.^76^ Even more intriguing are combinations of two WSSs, such as THF and MeTHF, where their synergy disrupts local structural ordering, allowing ionic conductivity to increase at low temperatures without compromising the weakly solvated nature of the electrolyte. Such systems support stable Na plating/stripping at −40 °C and high current density (2 mA cm^−2^), outperforming traditional THF–glyme mixtures across multiple metrics.^22^

Across all of these studies, WSEs in Na systems function as a modular platform to push Na toward more demanding use cases. Whether by suppressing solvent–ion interactions, enhancing anion coordination, or modulating interface-local solvation dynamics, these approaches make weak coordination a controllable design variable rather than an intrinsic feature.

### 3.3. WSEs for K metal batteries

K-based batteries, including both K-ion batteries and K metal batteries, are increasingly viewed as compelling alternatives to Li-based systems. The appeal lies in the natural abundance of K, its low standard reduction potential (−2.93 V *vs.* SHE), and its low Lewis acidity, which collectively confer high energy density and favorable transport kinetics. However, these benefits are counterbalanced by significant interfacial challenges. On the anode side, graphite hosts suffer from cointercalation and structural damage due to the large ionic radius of K^+^, while metallic K anodes are prone to dendrite growth and low CE due to unstable SEI formation. These interfacial instabilities are intimately tied to the solvation structure of the electrolyte. Conventional carbonate or ether solvents tend to coordinate strongly with K^+^, forming solvent-dominated solvation shells that lead to organic-rich, fragile interphases. A distinct strategy, built around WSEs besides LHCEs, is emerging to address these limitations by engineering anion-rich solvation environments that shift decomposition pathways and promote stable interphases. A representative example of this strategy is reported by Heng *et al.*, who designed a nonflammable fluorinated ether-based WSE consisting of 1 M KFSI in methyl 2,2,2-trifluoroethyl carbonate (FEMC)/FEC. This system exploits the inherently weak coordination ability of the fluorinated solvents to reduce K^+^–solvent binding and promote anion participation in the primary solvation shell. Through combined DFT, Raman, and MD simulations, the electrolyte is shown to exhibit a solvation environment dominated by CIPs and AGGs, with FSI^−^ ions directly coordinating K^+^. As a result, the SEI and CEI are both rich in inorganic species such as KF and K_2_SO_4_, leading to highly stable cycling. A KVPO_4_F cathode paired with this electrolyte retains 84.4% of its capacity over 1600 cycles at 4.95 V, and K metal shows reversible plating/stripping without dendritic failure.^77^

While the fluorinated WSE design demonstrates excellent interfacial modulation, other approaches have focused on restructuring the solvent network by using co-solvents to attenuate solvation strength. Chen *et al.* introduce diethoxy methane (DEM) as a partially and weakly solvating co-solvent to modify the coordination structure of a conventional KFSI/DME electrolyte, forming KFSI/DEM-DME. DEM exhibits low DN and limited chelation ability, which enables it to partially enter the solvation shell of K^+^ while weakening the strong interactions between DME and the cation. The resulting electrolyte, termed a partially WSE, shifts the solvation structure toward anion-rich clusters without resorting to high salt concentrations. This restructuring facilitates formation of thin and stable SEIs, reduces desolvation energy barriers, and suppresses K^+^–solvent cointercalation into graphite. The optimized formulation enables reversible K^+^ intercalation and deintercalation in graphite for over 1000 cycles and K metal plating/stripping with an average CE of 99.4% over 400 cycles.^78^

The use of intrinsically WSSs provides a streamlined route to WSE design by eliminating the need for co-solvent tuning. Feng *et al.* show that cyclic ethers such as THF and THP weaken K^+^–solvent interactions and promote the formation of anion-rich solvation structures, dominated by aggregated ion pairs (69% AGG in THF). This shift facilitates the generation of compact, inorganic-rich SEIs that stabilize graphite anodes, enabling a reversible capacity of 269 mAh g^−1^ and high CE (98.6%).^79^ In another work, Wang *et al.* employ 1,3-dioxane, a non-fluorinated cyclic ether with inherently weak coordination, as the sole solvent in a 1 M KFSI electrolyte. The resulting WSE exhibits anion-dominated solvation and forms an SEI with superior mechanical integrity, leading to a CE of 99.2% and stable K plating over 1300 hours. This electrolyte also enables full-cell cycling with a Prussian Blue cathode, maintaining 84.1% capacity after 100 cycles and resisting oxidation up to 4.83 V.^80^

## 4. WSEs for multivalent metal batteries

### 4.1. WSEs in Zn metal batteries

Zn metal batteries differ fundamentally from alkaline metal systems in both ionic character and electrolyte environment. As a divalent cation, Zn^2+^ interacts more strongly with coordinating species, resulting in tighter solvation and slower desolvation kinetics. Unlike Li^+^ or Na^+^, which operate primarily in organic electrolytes, Zn is compatible with water, enabling the use of aqueous electrolytes that are safer, less flammable, and more cost-effective. These advantages make Zn metal batteries particularly attractive for large-scale energy storage. However, the aqueous environment introduces unique challenges that do not arise in organic systems. The strong Zn^2+^–H_2_O interaction leads to sluggish ion desolvation and uneven Zn plating. In the context of WSEs, H_2_O is a highly coordinating, high-*ε* molecule that readily occupies the primary solvation shell of Zn^2+^, displacing anions and disrupting the anion-rich coordination environment essential for WSE functionality. Its extensive hydrogen-bonding network not only stabilizes solvent-separated Zn^2+^ species but also facilitates proton mobility, accelerating the hydrogen evolution reaction (HER). More critically, the redox potential of water overlaps with that of Zn/Zn^2+^, triggering the HER as a major parasitic process. This is exacerbated by the extensive hydrogen-bonding network in water, which facilitates proton mobility and accelerates interfacial instability. The result is rapid dendrite growth, interfacial corrosion, and poor cycling reversibility issues that are far more pronounced in aqueous Zn metal batteries than in non-aqueous Li or Na systems.

**Fig. 10:**
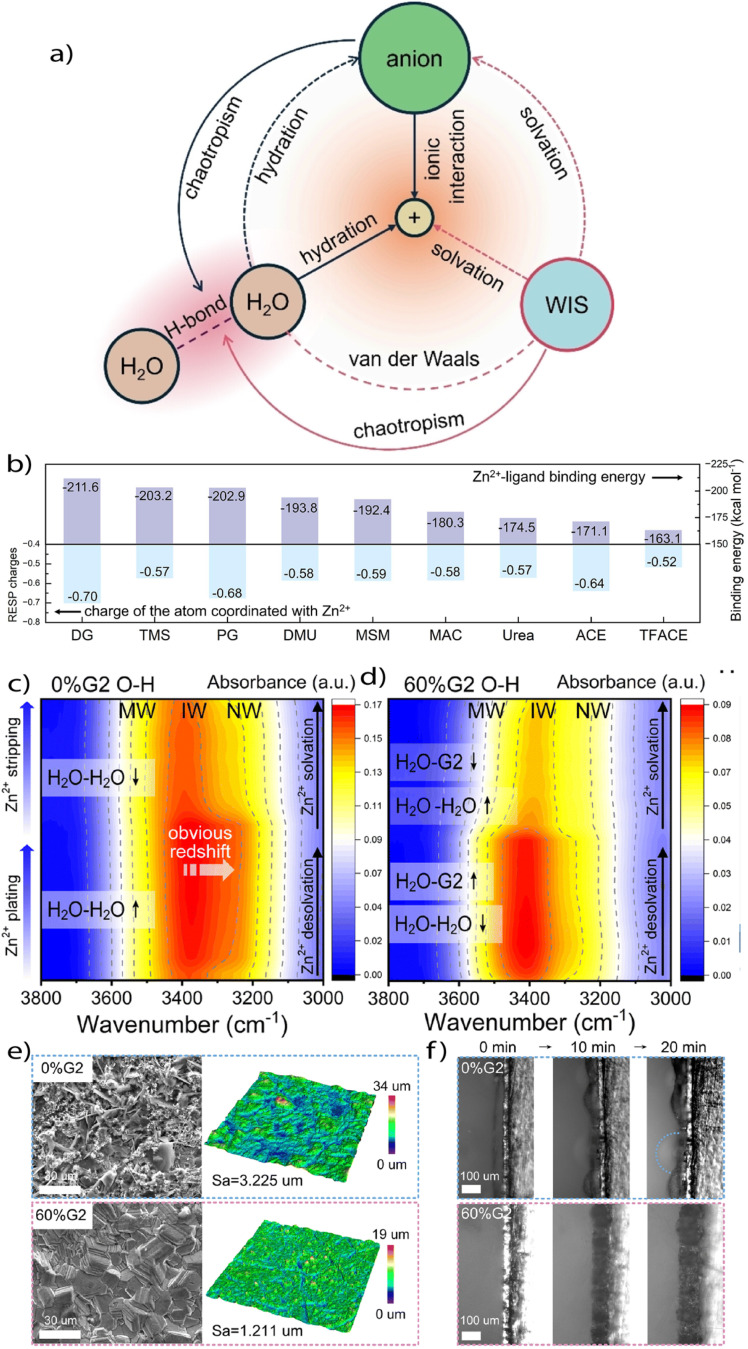
(a) A framework concluding important interactions between each two components in a weakly solvating aqueous electrolyte. Reproduced with permission.^81^ Copyright 2024, Wiley. (b) Comparison of Zn^2+^–ligand binding energies and the RESP charges of the atom coordinated with Zn^2+^ in different ligands obtained from DFT calculations. Reproduced with permission.^44^ Copyright 2025, Elsevier. Contour plots of *in situ* ATR spectra of electrolyte during Zn^2+^ plating/stripping process (corresponds to Zn^2+^ desolvation/solvation) with (c) 0%G2 and (d) 60%G2 electrolytes. (e) SEM images, 3D reconstruction images, and arithmetical mean height (*S*_a_) values of Zn electrodes cycled in 0%G2 and 60%G2 electrolytes for 20 times at 1 mA cm^−2^, 1 mAh cm^−2^. (d) *In situ* optical microscopy observations of Zn deposition in 0%G2 and 60%G2. Reproduced with permission.^41^ Copyright 2024, Royal society of chemistry.

Because of this complexity, conventional electrolyte strategies are often insufficient for Zn batteries. In response, WSEs have emerged as a targeted approach to mitigate Zn-specific interfacial degradation. Rather than relying solely on salt concentration or additives, WSEs aim to restructure the solvation shell by reducing Zn^2+^–H_2_O coordination, increasing anion participation, and disrupting the hydrogen-bonding network of water. These effects lower the desolvation barrier, suppress the HER, and promote the formation of stable, anion-derived interphases. The design of WSEs in aqueous systems therefore addresses three critical levers: the strength of Zn^2+^ solvation, the nature of hydrogen bonding in the bulk electrolyte, and the identity of anions participating in interfacial chemistry (Fig. 10a). Together, these principles define a platform for controlling interfacial reactivity at the molecular level, one that is essential for enabling long-cycle, high-rate Zn metal batteries under aqueous conditions.^81^

Recent studies have demonstrated that each of these properties can be regulated independently or synergistically through electrolyte design. One approach is to weaken Zn^2+^–ligand interactions by introducing coordinating species with inherently low donor strength. Xu *et al.* employed trifluoroacetamide (TFACE) in a hydrated eutectic system, where the low binding affinity between TFACE and Zn^2+^ permitted TFSI^−^ anions to dominate the solvation environment (Fig. 10b). This restructuring facilitated the formation of an anion-derived SEI and enabled long-term stability in Zn‖PANI (polyaniline) full cells over 3000 cycles.^44^ In parallel, Wu and Liu proposed a descriptor-based framework to rationalize the selection of secondary solvents that disrupt water's hydrogen-bond network without introducing new coordination pathways to Zn^2+^. Solvents such as AN and sulfolane, with low hydrogen bond donor and acceptor strengths, were shown to weaken proton transport and reduce water reactivity while preserving ionic mobility. Their analysis clarifies that effective WSE design is not solely about solvating power, but also about selecting solvents that act as hydrogen-bond “breakers” rather than “builders” in the aqueous matrix.^81^

A particularly insightful demonstration of aqueous WSE design comes from Zhang *et al.*, who introduced a hybrid electrolyte using diethylene glycol dimethyl ether (G2) as a soft co-solvent to modulate Zn^2+^ solvation without excessively increasing desolvation barriers. The moderate DN (19 kcal mol^−1^) and low *ε* (7.4) of G2 effectively displaced water from the Zn^2+^ solvation sheath, enhanced Zn^2+^–anion (OTf^−^) interactions, and enabled the formation of a robust anion-derived SEI composed of ZnS–ZnSO_3_–ZnF_2_ (Fig. 10c and d). This system not only demonstrated highly reversible Zn plating/stripping over 7500 h but also enabled exceptional cycling stability under extreme temperatures (−45 to 60 °C). By simultaneously restructuring hydrogen-bond networks and suppressing parasitic H_2_O reduction, the G2-based WSE exemplifies how soft co-solvents can reconcile interfacial stability, desolvation kinetics, and wide-temperature operability in aqueous Zn metal batteries. Moreover, the electrolyte modulates Zn deposition morphology by promoting horizontal crystal orientation, reducing nucleation overpotential, and increasing SEI resistivity, collectively suppressing dendritic growth during prolonged cycling (Fig. 10e and f).

An alternative strategy focuses on anion-centered solvation control by tuning the electron-donating characteristics of co-ligands. Chen *et al.* demonstrated that increasing the methyl substitution in urea-based eutectic ligands decreases their ability to coordinate Zn^2+^, thereby increasing the relative participation of Cl^−^ anions in the solvation sheath. This ligand-driven shift in solvation structure led to more homogeneous Zn deposition and improved interfacial stability in Zn‖NaV_3_O_8_ full cells.^43^

Integrating strategies such as weakening Zn^2+^-solvent coordination, disrupting hydrogen bonding networks, and promoting anion-centered solvation, Xu *et al.* outlined a broader design landscape for aqueous WSEs, emphasizing that optimal performance arises when solvent polarity, DN, and anion identity are tuned to favor CIP formation without compromising salt dissociation or electrolyte conductivity. Their analysis underscores the need to engineer solvation environments where Zn^2+^ is neither fully hydrated nor fully solvent-separated, but instead coordinated in a mixed anion–solvent structure that accelerates desolvation while minimizing side reactions.^82^

Together, these studies establish that aqueous WSEs are not defined by a single solvent or salt, but by the deliberate tuning of local solvation environments to redirect interfacial chemistry. By weakening cation–solvent interactions, disrupting hydrogen bonding, and promoting targeted anion coordination, WSEs offer a route to overcome the fundamental challenges of aqueous zinc batteries and expand their practical viability.

While aqueous WSEs have made significant strides, nonaqueous systems offer a fundamentally different pathway for Zn anode stabilization, particularly by eliminating water-induced side reactions altogether. In contrast to aqueous environments, where HER and strong Zn^2+^–H_2_O interactions dominate interfacial failure, nonaqueous electrolytes enable control over solvation through the use of weakly coordinating solvents and highly dissociative anions. Cheng *et al.* introduced a nonaqueous WSE based on 1-methylimidazole (EMI) and Zn(TFSI)_2_, wherein the Zn^2+^ solvation shell consists of approximately five EMI molecules and one TFSI^−^ anion. This coordination motif promotes a high fraction of anion participation and results in the formation of a robust, inorganic-rich SEI (with ZnF_2_, ZnS, ZnN_*x*_), enabling ultra-stable Zn plating/stripping over 7900 h with an overpotential as low as 20 mV.^83^ Molecular dynamics and spectroscopic studies confirmed that the weak Zn–N interaction facilitates rapid desolvation, while the embedded TFSI^−^ anions modulate interfacial reactivity.

The benefits of nonaqueous WSEs extend beyond stability. Xu *et al.* emphasize that in fully nonaqueous systems, Zn^2+^ acts as the exclusive charge carrier, simplifying interfacial processes and reducing parasitic reactivity compared to aqueous analogues.^84^ Yet, challenges persist because organic solvents often suffer from low ionic conductivity, high viscosity, and compatibility constraints with cathode materials. Nonetheless, recent efforts employing imidazoles, acetamide-based eutectics, and solvent mixtures with high donor number, low coordinating power, and thermal robustness are beginning to redefine what nonaqueous Zn WSEs can achieve.

Collectively, these nonaqueous strategies do not replace aqueous WSEs but complement them—offering a parallel route to stabilize Zn anodes under extreme conditions, high voltages, or prolonged lifetimes. As such, the field is rapidly moving toward hybrid or dual-salt systems where aqueous and nonaqueous design principles can be strategically combined.^85^

### 4.2. WSEs in Mg metal batteries

WSEs have recently shown considerable promise in addressing the interfacial instability of Mg metal anodes, a longstanding issue driven by the high charge density of Mg^2+^ and its strong coordination to solvent molecules. Traditional solvents such as ethers or amines often form tightly bound solvation shells, which hinder desolvation and promote continuous solvent decomposition during cycling. By intentionally weakening the Mg^2+^–solvent interaction, WSEs help regulate interfacial chemistry, promote stable SEI formation, and improve reversibility.

A key strategy involves tuning both solvent coordination ability and salt dissociation behavior to favor CIP formation in the primary solvation shell. Amine-based electrolytes using 3-methoxypropylamine (MPA) and Mg(OTF)_2_ exemplify this approach: the electrolyte forms a CIP-dominated solvation structure that suppresses amine dehydrogenation and solvent degradation, yielding a CE of 99.6% for over 800 cycles. In contrast, fully dissociated systems like 2-methoxyethylamine (MEA)-TFSI exhibit rapid capacity fade due to hydrogen evolution and the dissolution of solvent reduction products, which fail to accumulate in the SEI. Spectroscopic and DFT analyses confirm that anion incorporation stabilizes the N–H bonds of coordinated amines, mitigating parasitic reactions and promoting interfacial passivation.^24^

In parallel, co-solvent approaches using weakly coordinating additives such as THF have proven effective in halogen-free Mg electrolytes. Adding small amounts of THF to Mg(NO_3_)_2_-based systems reorganizes the solvation environment, reduces overpotentials, and enhances Mg^2+^ mobility by lowering desolvation barriers. These THF-modified electrolytes demonstrate smoother Mg deposition, improved Mg–S cell lifetime, and reduced polysulfide shuttling. Though the absolute capacity is modest, the extended cycle life compared to THF-free systems highlights the benefits of weaker solvation in suppressing interfacial degradation and stabilizing both electrodes.^52^

Collectively, these studies illustrate how WSEs, either through intrinsic solvent selection or ion-pair engineering, can regulate solvation structure to enhance cathodic stability and Mg deposition reversibility. Nonetheless, their typically low ionic conductivity remains a limitation, necessitating future work to balance weak solvation with transport performance.

## 5. Summary and perspective

WSEs mark a paradigm shift in electrolyte design by repositioning solvation structure, not conductivity, viscosity, or salt concentration, as the central design constraint. By deliberately suppressing cation–solvent coordination through solvent-level properties such as low DN, low *ε*, moderate ESP_min_, and restricted steric accessibility, WSEs enable a fundamental restructuring of the solvation shell in favor of anion coordination. This reconfiguration is not a secondary effect but the governing principle behind enhanced interfacial stability, lower desolvation barriers, and reduced dendritic growth in reactive metal battery systems.

Mechanistically, WSEs operate by manipulating electrostatic interactions at the molecular scale. In conventional electrolytes, strong ion–dipole interactions dominate; solvent molecules stabilize cations by aligning their electron-rich donor atoms with the cationic field. This leads to tightly bound, solvent-dominated solvation shells and SSIPs, particularly for highly charged or small-radius ions such as Li^+^ and Mg^2+^. In contrast, WSEs suppress these dipolar interactions, allowing ion–ion coulombic attractions, especially cation–anion interactions, to govern solvation shell formation. The resulting anion-rich solvation structures directly alter the energetics and kinetics of desolvation at the electrode interface. Desolvation becomes less energy-intensive, improving charge-transfer efficiency and facilitating dense, uniform metal plating.

This change in coordination environment has direct implications for dendrite suppression. In solvent-rich electrolytes, reduction typically initiates at the solvent itself, forming unstable, organic-rich SEIs that fail to uniformly passivate the electrode surface. This promotes heterogeneous current distribution and dendritic growth. In WSEs, by contrast, the dominance of anions in the primary solvation shell ensures that interfacial reduction is anion-driven. The decomposition products, LiF, Na_3_PO_4_, ZnSO_4_, and related inorganic phases, form SEIs that are mechanically robust, electronically insulating, and ionically conductive. These interphases not only suppress dendrite nucleation but also enable reversible cycling at high current densities and low temperatures.

Across battery chemistries, the WSE framework has demonstrated significant versatility. In Li metal systems, rational solvent design strategies—such as partial fluorination, siloxane substitution, or dipole-depletion, have yielded electrolytes that simultaneously lower desolvation energy, stabilize high-voltage cathodes, and eliminate the need for salt superconcentration. Na metal systems, benefiting from Na^+^’s lower charge density and weaker electrostatic binding, more readily support weak solvation. Here, WSEs have expanded the operational window to −40 °C and beyond, while improving SEI uniformity and reducing side reactions. In aqueous Zn batteries, the role of electrostatic control becomes even more apparent: by weakening Zn^2+^–H_2_O binding and disrupting the hydrogen-bonding network, aqueous WSEs inhibit parasitic hydrogen evolution, favor Zn^2+^–anion coordination, and enable compact Zn deposition with long-term cycling stability.

Despite this progress, weak solvation remains underutilized in several chemistries. In K metal batteries, despite K^+^’s inherently low Lewis acidity and weak solvation tendency, has not seen systematic application of WSE design principles. Current efforts rely on empirical co-solvent tuning rather than descriptor-driven approaches to modulate electrostatics or desolvation behavior. Mg systems, in contrast, present the opposite challenge. Mg^2+^ exhibits strong electrostatic affinity for donor-rich solvents, resulting in high desolvation penalties and sluggish interfacial kinetics. While initial studies using amine- or THF-based electrolytes have demonstrated feasibility, a more rigorous understanding of how to modulate solvation geometry and interfacial reactivity in Mg-based WSEs is urgently needed. For trivalent systems like Al, strong solvent coordination and hydrolysis sensitivity make WSE implementation particularly complex, likely requiring the discovery of entirely new solvent families or diluents.

Methodologically, WSE research also faces several limitations. There is currently no unified electrostatic or solvation energy threshold to define weak coordination across chemistries. Most studies rely on heuristic solvent selection or qualitative solvation analysis. Furthermore, the trade-off between weak solvation and ionic transport remains unresolved. As solvents become less coordinating, conductivity and wettability tend to suffer. Several promising solutions, including mixed-solvent systems, entropy-driven solvation disorder, and asymmetric coordination environments, have been proposed, but a general strategy to balance solvation suppression and transport enhancement remains elusive.

Looking forward, WSEs offer more than an electrolyte formulation, they provide a mechanistic philosophy for interfacial engineering. To fully exploit this framework, future research must integrate solvation energetics, electrostatic design, and bulk transport behavior into a cohesive, descriptor-guided platform. Predictive models incorporating DN, *ε*, ESP_min_, and solvation energy should be coupled with machine learning or high-throughput screening to accelerate solvent discovery. At the same time, experimental tools capable of resolving local solvation environments, such as Raman, DOSY-NMR, XPS, and synchrotron imaging, must be deployed systematically across systems to validate and refine design rules. Cation-specific coordination criteria will be essential for extending WSEs beyond Li and Na to multivalent and aqueous systems, each of which imposes distinct electrostatic and interfacial constraints.

Ultimately, WSEs are not defined by their conductivity, volatility, or viscosity, but by how they control electrostatic interactions at the electrode–electrolyte interface. By enforcing a solvation environment that favors anion coordination and inorganic interphase formation, WSEs offer a scalable route to dendrite suppression, desolvation control, and long-term cycling stability. As battery technologies move toward more reactive chemistries and harsher operating conditions, the principles of weak solvation will become increasingly central to the design of next-generation electrolytes.

Practical considerations and scalability will play a decisive role in translating WSE concepts from laboratory studies to devices. LGEs, though enabling ultrawide temperature operation (−60 to +55 °C) and high Li^+^ transference numbers, require heavy pressurized containers to maintain the liquefied state, which lowers gravimetric energy density and introduces complexity in packaging and safety. Beyond LGEs, many WSE formulations rely on heavily fluorinated solvents or salts (FSI^−^, TFSI^−^, PF_6_^−^ derivatives). These materials present cost and sourcing challenges due to complex fluorine chemistry, and their hydrolysis sensitivity poses both performance and safety risks. Moreover, waste management of fluorinated byproducts raises environmental and ESG (Environmental, Social, and Governance) concerns, especially as global regulations tighten around persistent fluorinated compounds. Addressing these issues will require not only solvation-centric design but also consideration of synthetic scalability, cost, recyclability, and environmental impact. Developing fluorine-efficient or fluorine-free weakly solvating motifs, or hybrid systems that reduce reliance on exotic components, may thus be essential for future deployment.

## Author contributions

All of the authors contributed to the manuscript preparation. W. X. and M. K. conceived the outline of the manuscript. M. K. and W. X. wrote the original draft of the manuscript. K. Y., Y. S. M. and Y. S. discussed and revise the manuscript.

## Conflicts of interest

There are no conflicts to declare.

## Data Availability

No primary research results, software or code have been included and no new data were generated or analysed as part of this review.
